# Efficacy of intravenous thrombolysis in patients with mild acute ischemic stroke and large vessel occlusion with different hypoperfusion volumes

**DOI:** 10.1007/s10072-025-08109-7

**Published:** 2025-03-19

**Authors:** Kechun Chen, Min Liu, Wenjun Zhang, Zhengduo Li, Huimin Shi

**Affiliations:** Department of Neurology, Zhangjiagang Hospital Affiliated to Soochow University, Zhangjiagang, China

**Keywords:** Intravenous thrombolysis, Dual antiplatelet therapy, Mild acute ischemic stroke, Hypoperfusion volume of CT, Prognosis

## Abstract

**Objectives:**

To investigate early treatment strategies for patients with mild acute ischemic stroke and large vessel occlusion, we evaluated the efficacy of intravenous thrombolysis in patients with mild stroke and large vessel occlusion in different hypoperfusion volumes.

**Methods:**

Cohort data of consecutive patients (October 2021 to June 2024) with anterior circulation large vessel occlusion mild stroke (NIHSS ≤ 5) with onset < 24 h were retrospectively analyzed. We compared the outcomes of patients treated with intravenous thrombolysis (IVT) and dual antiplatelet therapy (DAPT). The outcomes of patients on IVT and DAPT were then compared between the large-volume hypoperfusion group (> 65 ml) and the small-volume hypoperfusion group (≤ 65 ml) based on the hypoperfusion volume of CT perfusion (Tmax > 6s).

**Results:**

A total of 222 patients were enrolled in the study (62 IVT, 160 DAPT). In 111 patients with small-volume hypoperfusion, there was no statistically significant difference in the rate of good prognosis (mRS0-1) at 90 days, the rate of early neurological deterioration and the rate of intracranial hemorrhage between the IVT and DAPT groups (*P* > 0.05). In another 111 patients with large-volume hypoperfusion, the IVT group had a higher rate of good prognosis (mRS0-1) at 90 days (*OR*:3.639,*95%CI*:1.249 ~ 10.601, *P*:0.018) and a higher rate of intracranial hemorrhage (*OR*:11.029, *95%CI*:1.015 ~ 119.873, *P*:0.049) compared to the DAPT group.

**Conclusions:**

In mild acute ischemic stroke patients with large-volume hypoperfusion and large-vessel occlusion, IVT has a higher rate of intracranial hemorrhage but a higher proportion of excellent functional outcomes compared with DAPT. Further randomized controlled trials are needed.

## Introduction

Mild acute ischemic stroke (AIS), defined as a National Institutes of Health Stroke Scale.

(NIHSS) score of 0 to 5 [1]. Previous studies have shown that the majority of patients with mild stroke have better outcomes with pharmacological treatments such as intravenous thrombolysis (IVT) or dual antiplatelet therapy (DAPT) [2, 3]. However, some patients still experience deterioration in neurological function, leading to disability and even death, particularly those with large vessel occlusion [4]. On the optimal early treatment strategy for patients with mild acute ischemic stroke and large vessel occlusion [5, 6],The European Stroke Organisation/European Society for Minimally Invasive Neurotherapy Guidelines for Mechanical Thrombolysis in Acute Ischaemic Stroke state that EVT is recommended for patients with acute LVO whose initial presentation is a mild stroke in the following situations: significant disabling stroke (e.g. significant motor deficit, aphasia or hemianopsia) and symptomatic worsening despite intravenous thrombolysis. Therefore, in clinical practice, we have used aggressive pharmacological treatment for such patients. Salvage EVT should be performed if the patient experiences early neurological deterioration within 24 h. A meta-analysis of intravenous thrombolysis in patients with mild stroke found no statistically significant difference in prognosis between IVT and DAPT in mild stroke. However, IVT may be beneficial in improving the prognosis of mild strokes associated with large vessel occlusion, although there is an increased risk of intracranial hemorrhage [7]. It is imperative to be able to identify individuals at high risk of mild stroke with large vessel occlusion as early as possible in order to formulate appropriate therapeutic strategies. The use of CT-perfused severe hypoperfusion volume has been shown to predict prognosis in patients with mild stroke and large vessel occlusion [8]. Therefore, this study aims to evaluate the effect of IVT on the prognosis of mild stroke patients with different levels of severe hypoperfusion to provide a reference for therapeutic decision-making.

## Methods

### Study participants

We analyzed data from a cohort of 554 patients (October 2021 to June 2024) with acute large vessel occlusion stroke with onset < 24 h who were consecutively admitted to Zhangjiagang Hospital affiliated to Soochow University. 67 patients with posterior circulation stroke, 234 patients with severe stroke (NIHSS ≥ 6), and 11 patients with incomplete data were excluded, 20 patients with mild stroke underwent salvage endovascular therapy after hospital admission. Finally, 222 patients with acute mild anterior circulation stroke (NIHSS ≤ 5) combined with large vessel occlusion were finally included in the study (153 males and 69 females, mean age was 66.70 ± 13.79 years). We divided the group of 62 IVT cases and the group of 160 DAPT cases according to the treatment used during the first 24 h after admission. There was no statistically significant difference between the baseline characteristics of the two groups (*P* > 0.05) and they were comparable (Table 1).

**Table 1:** Comparison of baseline characteristics between the intravenous thrombolysis (IVT) and the dual antiplatelet therapy (DAPT) groups

Variables	All (*n*=222)	DAPT (*n=*160)	IVT (*n*=62)	t/χ2/Z value	*P* value
**Baseline characteristics**					
Age, (years, ±*s*)	66.70±13.79	66.72±13.24	66.63±15.27	0.046	0.963
Gender (female, n%)	69(31.1)	45(28.1)	24(38.7)	2.337	0.126
NIHSS on admission median (M, IQR)	3(2,5)	3(1,5)	3(2,5)	-0.798	0.425
Baseline MAP (mmHg, ±*s*)	107.8±12.7	108.4±13.3	106.3±11.1	1.127	0.261
ASPECTS at admission, median (M, IQR)	10(9,10)	10(9,10)	10(9,10)	-0.246	0.806
CBF<30%(ml, M,IQR)	1.5(0,7.0)	1.4(0,7.8)	1.9(0,5.2)	-0.175	0.861
Tmax>6s(ml, M,IQR)	65.3(29.3,127.3)	73.2(30.8,133.7)	50.7(23.6,89.8)	-1.644	0.100
HIR (M, IQR)	0.12(0,0.30)	0.09(0,0.29)	0.14(0,0.32)	-1.050	0.294
**Stroke risk factors n (%)**					
Hyperlipidemia	39(17.6)	30(18.8)	9(14.5)	0.553	0.457
Hypertension	165(74.3)	119(74.4)	46(74.2)	0.001	0.978
Diabetes	55(24.8)	43(26.9)	12(19.4)	1.356	0.244
Atrial fibrillation	46(20.7)	31(19.4)	15(24.2)	0.632	0.427
Smoking	91(40.9)	68(42.5)	23(37.1)	0.539	0.463
Drinking	57(25.7)	40(25.0)	17(27.4)	0.137	0.711
**Vascular occlusion site (n**,**%)**				3.197	0.362
Middle cerebral arteries	171(77.0)	122(76.3)	49(79.0)		
Anterior cerebral arteries	15(6.8)	9(5.6)	6(9.7)		
Internal carotid artery	36(16.2)	29(18.1)	7(11.3)		
**Baseline laboratory tests**					
FBG(mmol/L, M,IQR)	5.84(5.05,7.11)	5.85(5.07,7.11)	5.75(4.93,7.11)	-0.448	0.654
HbA1C (*M*,* IQR*)	6.0(5.6,6.9)	6.0(5.6,7.1)	5.9(5.6,6.3)	-1.692	0.091
LDL-C(mmol/L; ±*s*)	2.73±0.97	2.78±1.02	2.60±0.84	1.267	0.206
NLR(*M*,* IQR*)	2.84(1.78,4.61)	2.84(1.78,4.68)	2.96(1.88,4.73)	-0.147	0.883
D-dimer (mmol/L, M, IQR)	0.39(0.23,0.84)	0.39(0.19,0.84)	0.38(0.24,0.72)	-0.662	0.508
time from onset to NCCT(min, M,IQR)	284(225,375)	289(226,387)	262(225,338)	-1.237	0.216
**hemorrhagic transformation (n**,**%)**				10.720	0.030
HI1	4(1.8)	1(0.6)	3(4.8)		
HI2	2(0.9)	1(0.6)	1(1.6)		
PH1	2(0.9)	0(0)	2(3.2)		
PH2	1(0.5)	1(0.6)	0(0)		
ASPECTS at 7d, (M, IQR)	8(7,9)	8(7,9)	8(7,9)	-0.196	0.844

ASPECTS, Alberta Stroke Project Early CT Score; CBF, Cerebral Blood Flow; FBG, Fasting blood glucose; HbA1C, Hemoglobin A1c; HIR, Hypoperfusion intensity ratio; LDL-C, Low-Density Lipoprotein Cholesterol; NLR, Neutrophil Lymphocyte Ratio; NIHSS, National Institutes of Health Stroke Scale

Inclusion criteria were (1) age ≥ 18 years, (2) time from onset to presentation < 24 h, CTA-confirmed large vessel occlusion of the anterior circulation (intracranial segment of the internal carotid artery or main trunks of the middle cerebral arteries M1 and M2 or main trunks of the anterior cerebral arteries A1 and A2), (3) baseline NIHSS ≤ 5. Exclusion criteria: (1) previous modified Rankin Scale (mRS) score ≥ 2, (2) incomplete follow-up data, (3) Bleeding tendency, such as platelets less than 100*109/l. (4) Patient underwent salvage endovascular therapy after hospital admission.

### Data collection and follow-up

Clinical data of patients were collected through an electronic medical record system. We collected clinical data on the enrolled patients, including demographics, stroke risk factors, NIHSS on admission, and blood pressure levels. We collected laboratory data including baseline glucose, glycosylated hemoglobin, LDL cholesterol, neutrophil/lymphocyte ratio, and D-dimer. We collected imaging data including Alberta Stroke Program Early CT Score (ASPECTS) on admission and ASPECTS of DWI at onset 7d, site of vessel occlusion, cerebral blood flow (CBF) < 30% volume representing the core of the infarct, time to peak (Tmax) > 6s volume representing severe hypoperfusion and hypoperfusion intensity ratio (HIR) representing the degree of collateral circulation: Tmax > 10s volume/Tmax > 6s volume ratio [9]. We divided the enrolled patients into a large-volume hypoperfusion group (Tmax > 6s volume > 65 ml) and a small-volume hypoperfusion group (Tmax > 6s volume ≤ 65 ml), based on the Tmax > 6s volume threshold for CT perfusion in a previous study [8]. All patients completed the 3-month follow-up. Primary endpoint: modified Rankin Scale (mRS) score at 90 days, good prognosis defined as (mRS 0–2) and excellent prognosis defined as (mRS 0–1). Secondary endpoints: intracranial hemorrhage (ICH) documented by (European Cooperative Acute Stroke Study)ECASS staging, HI was defined as petechial infarction without space-occupying effect: HI1 (small petechiae) and HI2 (more confluent petechiae). PH was defined as hemorrhage (coagulum) with mass effect: PH1 (30% of the infarct area with some mild space-occupying effect) and PH2 (30% of the infarct area with significant space-occupying effect or clot away from the infarct area) [10], Symptomatic intracranial hemorrhage was defined as a clinical deterioration of at least 4 points on the NIHSS [11], and early neurological deterioration (END) (defined as an increase of at least4 points in the 24-hour NIHSS from the NIHSS on admission [12]). All of the above data was collected by two brain center managers and neurologists who were unaware of the purpose and subgroups of the study.

CT Examinations protocol: At Zhangjiagang Hospital affiliated to Soochow University, all CT scans were performed on a 64-slice scanner (GE Revolution Ace). The imaging parameters for CTP were: tube voltage 100 kV, tube current 120 mAs, whole brain coverage in the z-axis, FOV 220*220 mm2, matrix 512*512, slice thickness 5 mm, and 14 consecutive phases acquired with a temporal resolution of 4s. A total of 50 ml of iodinated contrast was injected intravenously at a flow rate of 5 ml/s, followed by a 20 ml saline flush. CTA was performed 1 min after completion of CTP with the following parameters: tube voltage 120 kV, tube current 480 mAs, FOV 220*220 mm2, matrix 512*512, slice thickness 0.625 mm, and slice number 249. The same iodinated contrast agent was injected intravenously at the same flow rate as for CTP, and the contrast agent dose was 25 ml.

### Treatment

First 24-hour treatment: Patients with indications for intravenous thrombolysis received intravenous thrombolysis with alteplase (0.9 mg/kg) and patients without indications for IVT received dual antiplatelet therapy with aspirin and clopidogrel. Treatment program after 24 h: In the IVT group, cranial CT was performed 24 h after IVT. If no intracranial hemorrhage was observed on CT, DAPT (aspirin and clopidogrel) was given for 90 days, and if intracranial hemorrhage was observed on CT, no antithrombotic treatment was given. In the DAPT group, all patients had DAPT (aspirin and clopidogrel) for 90 days. If a repeat cranial CT scan shows an intracranial hemorrhage, antiplatelet therapy is stopped. At the same time, we proactively managed the high-risk factors for stroke (blood pressure, blood lipids, blood glucose) and started rehabilitation training as early as possible.

### Statistical analysis

Independent samples t-test or Mann-Whitney U-test was used for continuous variables, and the chi-squared test or Fisher’s exact test was used for binary variables to perform univariable analyses as appropriate. Binary logistic regression was used to analyze the relationship between each group and prognosis, with correction factors being univariate analysis of *P* < 0.1 and clinically significant variables, and odds ratio (*OR*) and *95%CI* were calculated. All data were analyzed using the SPSS statistical package (version 27.0; SPSS, Chicago, IL, USA). *P* < 0.05 was considered a statistically significant difference. This study was approved by the Ethics Committee of Zhangjiagang Hospital affiliated to Soochow University (No. ZJGYYLL-2024-01-006). As this was a retrospective cohort study, individual informed consent was not required, but the data of all study patients were kept strictly confidential.

## Results

A total of 160 DAPT patients and 62 IVT patients were included. We compared the prognosis of the two groups at 90 days after adjustment for age, sex, baseline NIHSS, baseline ASPECTS, and baseline infarct core volume (CBF < 30% volume). There was no statistical difference between the two groups in the rates of good prognosis (mRS0-2), good prognosis (mRS0-1), or proportion of END. However, there was a higher rate of intracranial hemorrhage in the IVT group compared to the DAPT group (*OR*: 6.235, 95% CI: 1.427–27.235, *P* = 0.015), (Table 2).

**Table 2:** Comparison of outcomes between the DAPT and IVT groups

Outcomes	DAPT	IVT	Model 1		Model 2	
	(*n*=160)	(*n*=62)	OR(95%CI)	*P* value	OR(95%CI)	*P* value
90d mRS0-2	127(79.4)	51(82.3)	1.205(0.566~2.565)	0.629	1.224(0.554~2.704)	0.618
90d mRS0-1	79(49.4)	38(61.3)	1.623(0.893~2.951)	0.112	1.667(0.863~3.218)	0.128
END	33(20.6)	17(27.4)	1.454(0.739~2.860)	0.278	1.363(0.678~2.738)	0.384
ICH	3(1.9)	6(9.7)	5.607(1.357~23.176)	0.017	6.235(1.427~27.235)	0.015

Model 1: Uncorrected for all factors; Model 2: Correction factors (age, sex, baseline NIHSS, ASPECTS and CBF <30% volume)

Within the 111 large-volume hypoperfusion group, there was no statistically significant difference in the comparison of baseline characteristics between the 26 patients on IVT and the 85 patients on DAPT (Table 3). However, after adjustment for clinically relevant variables, we compared the prognosis of patients in the two groups and showed that the IVT group had a higher proportion of patients with a good prognosis (mRS0-1) at 90 days (*OR*:3.639, *95%CI*:1.249 ~ 10.601, *P*:0.018) and a higher rate of intracranial hemorrhage (*OR*:11.029, *95%CI*:1.015 ~ 119.873, *P*:0.049) compared to the DAPT group. (Table 4). Within the 111 small-volume hypoperfusion group, there was a statistically significant difference in the baseline NIHSS and hypoperfusion intensity ratio when comparing the baseline characteristics of 36 patients on IVT and 75 patients on DAPT (*P* < 0.05) (Table 5). After adjustment for baseline NIHSS, hypoperfusion intensity ratio, and clinically relevant variables, our comparison of prognosis between the two groups showed that the difference in the rate of good prognosis (mRS0-1) at 90 days, the rate of early neurological deterioration, and the rate of intracranial hemorrhage between the IVT group and the DAPT group was not statistically significant *(P* > 0.05) (Table 6).

**Table 3:** Comparison of baseline characteristics between the two large-volume hypoperfusion IVT and DAPT groups

Variables	All (*n*=111)	DAPT (*n=*85)	IVT (*n=*26)	t/χ2/Z value	*P* value
**Baseline characteristics**					
Age, (years, ±*s*)	67.81±14.47	66.89±14.76	70.81±13.33	-1.209	0.229
Gender (female, n%)	32(28.8)	23(27.1)	9(34.6)	0.554	0.457
NIHSS on admission median (M, IQR)	4(2,5)	4(2,5)	3.5(2,5)	-0.443	0.665
Baseline MAP (mmHg, ±*s*)	106.31±11.79	106.13±12.21	106.82±10.74	-0.251	0.802
ASPECTS at admission, median (M, IQR)	10(8,10)	10(8,10)	10(8,10)	-0.078	0.938
CBF<30%(ml, M,IQR)	3.9(0.4,11.8)	3.9(0.5,14.5)	2.3(1.2,6.2)	-0.774	0.439
Tmax>6s(ml, M,IQR)	127.3(84.4,174.0)	129.2(84.5,179.5)	98.5(81.0,156.8)	-1.017	0.309
HIR(M, IQR)	0.21(0.08,0.39)	0.22(0.08,0.41)	0.19(0.07,0.32)	-0.669	0.504
**Stroke risk factors n (%)**					
Hyperlipidemia	22(19.8)	16(18.8)	6(23.1)	0.227	0.634
Hypertension	82(73.9)	62(72.9)	20(76.9)	0.164	0.686
Diabetes	22(19.8)	19(22.4)	3(11.5)	1.465	0.226
Atrial fibrillation	23(20.7)	16(18.8)	7(26.9)	0.795	0.373
Smoking	41(36.9)	34(40.0)	7(26.9)	1.462	0.227
Drinking	28(25.2)	19(22.4)	9(34.6)	1.587	0.208
**Vascular occlusion site (n**,**%)**				2.741	0.433
Middle cerebral arteries	87(65.2)	65(77.4)	22(84.6)		
Anterior cerebral arteries	2(1.8)	1(1.2)	1(3.8)		
Internal carotid artery	21(18.9)	18(21.2)	3(11.5)		
**Baseline laboratory tests**					
FBG(mmol/L, M,IQR)	5.72(5.05,7.26)	5.72(5.06,7.30)	5.73(4.88,7.11)	-0.533	0.594
HbA1C (*M*,* IQR*)	6.0(5.6,6.7)	6.0(5.7,6.8)	5.8(5.5,6.2)	-1.589	0.112
LDL-C(mmol/L; ±*s*)	2.71±1.08	2.79±1.14	2.47±0.81	1.324	0.188
NLR(*M*,* IQR*)	2.81(1.84,4.74)	2.84(1.89,5.79)	2.39(1.61,3.77)	-1.149	0.251
D-dimer (mmol/L, M, IQR)	0.40(0.23,0.84)	0.45(0.19,0.91)	0.37(0.26,0.48)	-0.370	0.711
time from onset to NCCT(min, M,IQR)	284(226,366)	286(224,380)	272(234,331)	-0.512	0.609
**hemorrhagic transformation (n**,**%)**				7.548	0.056
HI1	2(1.8)	1(1.2)	1(3.8)		
HI2	1(0.9)	0	1(3.8)		
PH1	1(0.9)	0	1(3.8)		
PH2	0(0)	0	0(0)		
ASPECTS at 7d(M, IQR)	8(7,9)	8(7,9)	8(7,9)	-0.595	0.552

ASPECTS, Alberta Stroke Project Early CT Score; CBF, Cerebral Blood Flow; FBG, Fasting blood glucose; HbA1C, Hemoglobin A1c; HIR, Hypoperfusion intensity ratio; LDL-C, Low-Density Lipoprotein Cholesterol; NLR, Neutrophil Lymphocyte Ratio; NIHSS, National Institutes of Health Stroke Scale

**Table 4:** Comparison of outcomes between the two large-volume hypoperfusion IVT and DAPT groups

Outcomes	DAPT	IVT	Model 1		Model 2	
	(*n*=85)	(*n*=26)	OR(95%CI)	*P* value	OR(95%CI)	*P* value
90d mRS0-2	62(72.9)	20(76.9)	1.558(0.526~4.617)	0.424	1.205(0.404~3.597)	0.738
90d mRS0-1	31(36.5)	17(65.4)	3.290(1.310~8.263)	0.011	3.639(1.249~10.601)	0.018
END	24(28.2)	8(30.8)	1.130(0.434~2.943)	0.803	1.209(0.443~3.299)	0.711
ICH	1(1.2)	3(11.5)	10.957(1.088~11-0.347)	0.042	11.029(1.015~119.873)	0.049

Model 1: Uncorrected for all factors; Model 2: Correction factors (age, sex, baseline NIHSS, ASPECTS and CBF <30% volume)

**Table 5:** Comparison of baseline characteristics between the two small-volume hypoperfusion IVT and DAPT groups

Variables	All (*n*=111)	DAPT (*n*=75)	IVT (*n*=36)	t/χ2/Z value	*P* value
**Baseline characteristics**					
Age, (years, ±*s*)	65.59±13.06	66.53±11.36	63.61±16.03	1.105	0.272
Gender (female, n%)	37(33.3)	22(29.3)	15(41.7)	1.665	0.197
NIHSS on admission median (M, IQR)	3(1,4)	2(1,4)	3(2,4.5)	-2.078	0.038
Baseline MAP (mmHg, ±*s*)	109.4±13.5	111.0±14.1	105.9±11.6	1.896	0.061
ASPECTS at admission, median (M, IQR)	10(9,10)	10(9,10)	10(9,10)	-0.012	0.991
CBF<30%(ml, M,IQR)	0(0,2.6)	0(0,1.7)	1.4(0,4.2)	-1.670	0.095
Tmax>6s(ml, M,IQR)	29.3(13.8,46.0)	29.4(13.2,46.0)	28.5(13.8,45.2)	-0.031	0.975
HIR(M, IQR)	0(0,0.18)	0(0,0.09)	0.09(0,0.33)	2.808	0.005
**Stroke risk factors n (%)**					
Hyperlipidemia	17(15.3)	14(18.7)	3(8.3)	2.003	0.157
Hypertension	83(74.8)	57(76.0)	26(72.2)	0.184	0.668
Diabetes	33(29.7)	24(32.0)	9(25.0)	0.571	0.450
Atrial fibrillation	23(20.7)	15(20.0)	8(22.2)	0.073	0.787
Smoking	50(45.0)	34(45.3)	16(44.4)	0.008	0.930
Drinking	29(26.1)	21(28.0)	8(22.2)	0.421	0.517
**Vascular occlusion site (n**,**%)**				1.017	0.797
Middle cerebral arteries	83(74.8)	56(74.7)	27(75.0)		
Anterior cerebral arteries	13(11.7)	8(10.7)	5(13.9)		
Internal carotid artery	15(13.5)	11(14.6)	4(11.1)		
**Baseline laboratory tests**					
FBG(mmol/L, M,IQR)	5.86(5.07,7.11)	5.88(5.07,7.05)	5.75(4.98,7.26)	-0.290	0.772
HbA1C (*M*,* IQR*)	5.9(5.6,7.3)	6.1(5.6,7.5)	5.9(5.6,6.8)	-0.839	0.401
LDL-C(mmol/L; ±*s*)	2.74±0.85	2.77±0.85	2.69±0.85	0.478	0.634
NLR(*M*,* IQR*)	2.97(1.76,4.55)	2.66(1.71,3.89)	2.99(1.94,5.25)	-1.042	0.298
D-dimer (mmol/L, M, IQR)	0.39(0.19,0.81)	0.35(0.22,0.73)	0.48(0.23,1.62)	-1.236	0.217
time from onset to NCCT(min, M,IQR)	279(225,381)	300(231,392)	247(222,366)	-1.279	0.201
**hemorrhagic transformation (n**,**%)**				7.292	0.121
HI1	2(1.8)	0(0)	2(5.6)		
HI2	1(0.9)	1(1.3)	0(0)		
PH1	1(0.9)	0(0)	1(2.8)		
PH2	1(0.9)	1(1.3)	0(0)		
ASPECTS at 7d, (M, IQR)	8(8,9)	9(8,9)	8(7,9)	-1.422	0.155

ASPECTS, Alberta Stroke Project Early CT Score; CBF, Cerebral Blood Flow; FBG, Fasting blood glucose; HbA1C, Hemoglobin A1c; HIR, Hypoperfusion intensity ratio; LDL-C, Low-Density Lipoprotein Cholesterol; NLR, Neutrophil Lymphocyte Ratio; NIHSS, National Institutes of Health Stroke Scale

**Table 6:** Comparison of outcomes between the two small-volume hypoperfusion IVT and DAPT groups

Outcomes	DAPT	IVT	Model 1		Model 2	
	(*n*=75)	(*n*=36)	OR(95%CI)	*P* value	OR(95%CI)	*P* value
90d mRS0-2	65(86.7)	30(83.3)	0.769(0.256~2.313)	0.640	0.948(0.275~3.268)	0.932
90d mRS0-1	48(64.0)	21(58.3)	0.788(0.349~1.776)	0.565	0.867(0.326~2.311)	0.776
END	9(12.0)	9(25.0)	2.444(0.876~6.825)	0.088	2.508(0.770~8.165)	0.127
ICH	2(2.7)	3(8.3)	3.318(0.529~20.806)	0.200	3.572(0.346~36.829)	0.285

Model 1: Uncorrected for all factors; Model 2: Correction factors (age, sex, baseline NIHSS, ASPECTS, HIR and CBF <30% volume)

## Discussion

In this study, we found no significant difference in outcomes between patients treated with IVT and DAPT (after 24 h) versus DAPT in patients with mild stroke caused by occlusion of large anterior circulation vessels. However, the IVT group had a higher rate of intracranial hemorrhage. While IVT was associated with a higher rate of good prognosis than DAPT in patients with large-volume hypoperfusion, there was no significant effect of either treatment on patient prognosis in patients with small-volume hypoperfusion.

About 20% of patients with acute ischemic mild stroke experience early neurological deterioration [13]. A higher percentage of these patients with combined large vessel occlusion experienced neurological deterioration [14]. 50/222 (22.5%) of the patients enrolled in this study developed END within 24 h. This is similar to previous studies [13]. A study in an Asian population found that older age, high baseline NIHSS, high baseline glucose and lack of intravenous thrombolysis were associated with END [15], Poor collateral circulation is also strongly associated with the development of END in such patients [16]. In patients with large-vessel occlusive mild stroke, current guidelines do not recommend endovascular intervention and salvage EVT may be performed in patients with early neurological deterioration within 24 h. However, previous studies of early therapeutic measures such as IVT or DAPT in large-vessel occlusive mild stroke had not found a significant impact on patient prognosis [17, 18]. Studies have shown that the use of perfusion imaging to guide reperfusion therapy, including intravenous thrombolysis and mechanical thrombectomy, has good results in severe ischemic stroke [19, 20]. The use of neuroimaging to study the prognosis of mild stroke with large vessel occlusion has shown a strong association with collateral circulation. The location of the diseased vessel, the aetiology of the stroke and different pathogenetic mechanisms may lead to different degrees of collateral compensation in patients [21]. The perfusion parameter HIR is used to assess the collateral compensatory capacity of brain tissue in patients. Higher HIR values have been shown to be associated with a poorer clinical prognosis. This is indicative of more severe tissue damage and a reduced likelihood of brain tissue salvage by reperfusion [22]. Consequently, in the present study, patients in the large-volume hypoperfusion group had a significantly higher HIR than those in the small-volume hypoperfusion group, indicating the presence of unstable collateral compensation. This group of patients is at greater risk of adverse outcomes. According to the Wang study [8], We used the Tmax > 6s threshold to compare the prognostic effect of IVT and DAPT groups. We found that in patients with large-volume hypoperfusion (> 65 ml), IVT had a higher proportion of good prognosis compared with DAPT, IVT increased the risk of intracranial hemorrhage, which was similar to previous findings [13]. However, in our study, asymptomatic intracranial hemorrhage was more common in patients with large-volume hypoperfusion. We observed that the difference in outcome between the two treatments in patients with high-volume hypoperfusion, whereas in patients with small-volume hypoperfusion (< 65 ml), there was no significant difference in patient prognosis between IVT and DAPT. It is possible that in patients with large-volume hypoperfusion, effective collateral circulation is more likely to deteriorate, and the diseased brain tissue suffers from poor oxygen delivery and local metabolic disturbances due to thrombosis as a result of slowed blood flow [23]. IVT acts not only on large vessel lesions but also on distal collateral vessels, and IVT has a high recanalisation rate for fibrinogen-dominant white or mixed thrombi compared with dual antiplatelet therapy [24]. However, compared to Wang’s study, we included patients with a relatively low rate of intravenous thrombolysis, and we also set the END time to within 24 h instead of 72 h. In the present study, we used DAPT for a longer period of time (90 days) in both groups of patients, which may differ from previous studies. In addition, the reason for the difference in outcome between the two treatments in patients with high-volume hypoperfusion may be related to infarct volume and infarct location, but we also found no difference when comparing ASPECTS of DWI at 7 days after onset, and we believe that different infarct locations may have a greater impact on patient prognosis. In contrast, in patients with small volume hypoperfusion, adequate collateralization and low distal thrombus burden, there was no significant difference in patient prognosis between IVT and DAPT. Therefore, selective intravenous thrombolysis guided by perfusion imaging should be used in mild stroke to improve the good prognosis rate and reduce the poor prognosis such as intracranial hemorrhage. In such patients, this is an important reference point for early treatment decisions.

There are some limitations to this study, such as the lack of complete follow-up data on large intracranial vessel occlusion, and a proportion of patients who underwent further mechanical thrombectomy due to early neurological deterioration were not included in this study. Our study did not address the different locations of infarct foci for further categorisation, and data on final infarct volume were lacking. Therefore, caution should be exercised in interpreting the study results. In addition, the limited sample size of a single-centre study may lead to biased results. We look forward to a prospective, large trial of neuroimaging guided reperfusion therapy for large vessel occlusion in mild stroke to validate our findings.

In conclusion, in patients with mild ischemic stroke with large-vessel occlusion and large-volume hypoperfusion, intravenous thrombolysis is associated with a higher rate of good 90-day outcome than dual antiplatelet therapy. However, in patients with small-volume hypoperfusion, there was no difference between intravenous thrombolysis and dual antiplatelet therapy.

## Data Availability

The datasets generated during and/or analyzed during the current study are not publicly available, but are available from the corresponding author on reasonable request.
